# Rapid High-Sensitivity Detection of Antibodies to *Trypanosoma cruzi* With a Recombinant Tc24 Antigen-Based Magneto-Immunoassay: A Pilot Study

**DOI:** 10.1093/infdis/jiaf123

**Published:** 2025-03-06

**Authors:** Kavya L Singampalli, Bin Zhan, Rojelio Mejia, Peter B Lillehoj

**Affiliations:** Department of Bioengineering, Rice University; Medical Scientist Training Program, Baylor College of Medicine; Department of Pediatrics–Tropical Medicine, Baylor College of Medicine; Department of Pediatrics–Tropical Medicine, Baylor College of Medicine; Department of Bioengineering, Rice University; Department of Mechanical Engineering, Rice University, Houston, Texas

**Keywords:** Chagas disease, diagnostic, immunoassay, Tc24, *Trypanosoma cruzi*

## Abstract

The diagnosis of chronic Chagas disease is challenging due to the wide genetic diversity of *Trypanosoma cruzi*, the causative agent of Chagas disease, and low levels of parasitemia, resulting in low sensitivity and accuracy with existing diagnostics. We report a magneto-immunoassay that employs dually labeled magnetic beads incorporating a recombinant Tc24 antigen, which is homologous across multiple discrete typing units of *T cruzi*. In this pilot study, 102 serum samples from 7 endemic countries were tested by this magneto-immunoassay, revealing its ability to distinguish Chagas-positive from Chagas-negative cases more accurately and faster than a standard Tc24-based immunoassay.

## Teaser

We developed a Tc24-based magneto-immunoassay for high-sensitivity detection of anti–*Trypanosoma cruzi* IgG. Clinical serum samples from multiple Chagas-endemic countries were tested, revealing its ability to distinguish Chagas-positive from Chagas-negative cases more accurately and faster than a standard Tc24-based immunoassay.

Chagas disease (CD), or American trypanosomiasis, is a neglected tropical disease caused by the parasite *Trypanosoma cruzi* that affects approximately 6 to 7 million people worldwide, mostly in Latin America [1]. CD has acute and chronic phases, both of which can have subclinical symptoms, complicating clinical diagnosis [2]. If left untreated, acute infection progresses to the chronic indeterminate phase, in which ∼30% of patients develop life-threatening complications, including cardiac (cardiomyopathy, heart failure, cardiac arrest) or gastrointestinal conditions (megaesophagus, megacolon) [1].

Methods for diagnosing acute CD include microscopic identification of *T cruzi* in blood smears or the detection of *T cruzi* DNA with nucleic acid–based tests. During the chronic phase, the level of parasitemia drops below the limit of detection of polymerase chain reaction (PCR)–based tests, resulting in low sensitivity. Thus, the diagnosis of chronic infection relies mainly on serologic assays, such as indirect immunofluorescence, indirect hemagglutination, and enzyme-linked immunosorbent assay (ELISA) [3]. There are currently four Food and Drug Administration–cleared serologic tests for the detection of chronic CD, which use crude parasitic antigens or a combination of recombinant antigens [4]. However, the accuracy of these and other tests is highly variable for the detection of different *T cruzi* discrete typing unit (DTU) subtypes. For example, one study found that commercial Chagas tests missed up to 12% of Chagas-seropositive cases from Argentina and up to 70% of the cases from Mexico [5], demonstrating the geographic variability of test performance. Additionally, the results of 2 independent tests can be discordant [6]. For these reasons, the current World Health Organization recommendations include using at least 2 serologic tests to confirm the diagnosis of chronic CD, leading to significant delays in diagnosis and excessive resource burden on the health care system [7].

Various approaches have been reported to improve the accuracy of serologic tests for chronic CD, including the development of novel recombinant/chimeric antigens to be used as capture proteins for *T cruzi* immunoassays [8, 9] and alternative diagnostic algorithms [10]. While these methods have resulted in improved sensitivity and specificity, they require long incubation periods (>3–4 hours) or were not evaluated for their ability to detect all 6 *T cruzi* DTUs [5]. The objective of this study was to evaluate the performance of a magneto-immunoassay prototype in detecting anti–*T cruzi* IgG in clinical serum samples from multiple Chagas-endemic countries and compare its performance against a standard Tc24-based ELISA. This assay employs dually labeled magnetic beads (DMBs) to enhance the capture of anti–*T cruzi* IgG in the sample and accelerate the transport of DMB–*T cruzi* IgG conjugates, resulting in enhanced analytical performance. DMBs are abundantly coated with horseradish peroxidase (HRP), an enzymatic reporter, and a recombinant Tc24 antigen that is highly specific to *T cruzi* [11]. Tc24 is 97% homologous across all *T cruzi* DTU subtypes and ubiquitously expressed in all stages of the *T cruzi* life cycle, making it an ideal capture protein for the detection of anti–*T cruzi* IgG [12, 13]. The performance of the magneto-immunoassay was evaluated by testing 60 human serum samples obtained from multiple Chagas-endemic countries in Latin America (Ecuador, Guatemala, Honduras, Mexico, Colombia, and Brazil) encompassing the 6 *T cruzi* DTUs, which revealed its ability to distinguish Chagas-positive and Chagas-negative cases in <1 hour with better accuracy than a standard Tc24-based ELISA.

## MATERIALS AND METHODS

### Magneto-Immunoassay Protocol

In this study, 96-well plates (3361; Corning) were incubated with 45 µL per well of 10-µg/mL anti–human IgG (109-005-190; Jackson Laboratories) overnight at 4 °C at 150 rpm to coat the wells. The plates were washed 5 times with 0.05% Tween 20 in phosphate-buffered saline (PBST), blocked with 3% bovine serum albumin (BSA) at room temperature for 1 hour, and dried at 4 °C for at least 8 hours before use. Furthermore, 1 mg of 200-nm-diameter carboxyl-functionalized magnetic beads (Ademtech) were bound to 2.5 µg of recombinant Tc24 and 300 µg of HRP by carbodiimide crosslinking. Briefly, the carboxyl sites on the magnetic beads were activated with 10 mg/mL of N-hydroxysuccinimide (130672; Millipore Sigma) and 1-ethyl-3-(3-dimethylaminopropyl)-carbodiimide (PG82079; Thermo Scientific) in 25mM MES buffer (2-[N-morpholino]ethanesulfonic acid, pH 5.0, 28390; Thermo Scientific). The beads were washed and incubated with equal volumes of HRP (6 mg/mL) and Tc24 (50 μg/mL) in MES buffer overnight (protected from light) at 500 rpm, followed by a 1.5-hour incubation with 3% BSA. The conjugated magnetic beads were stored in 400 µL of Stabilzyme HRP Stabilizer (SZ02; Surmodics Inc) at 4 °C (protected from light) until use.

To run the magneto-immunoassay, 100 µL of patient serum diluted at 1:800 in phosphate-buffered saline was incubated with 5 µL of DMBs for 20 minutes. The DMBs were then separated from solution, washed, and reconstituted to 100 µL in MatrixGuard assay diluent (SM02; Surmodics Inc). 85 µL of the DMB solution was added to an IgG-coated well and incubated for 14 minutes, followed by a 1-minute concentration on a custom magnetic stage. After a 5-minute incubation, the plate was washed 5 times with 0.05% PBST, and 100 µL of TMB substrate (3,3′,5,5′-tetramethylbenzidine, 308175; Neogen) was added. The reaction was stopped after 10 minutes using 2N sulfuric acid (50 µL), and the absorbance values were read by an Epoch plate reader (BioTek) at a 450-nm wavelength.

### Standard ELISA Protocol

The standard Tc24-based ELISA was performed with Immulon4 HBX 96-well plates (3855; Thermo Fisher). Recombinant Tc24 (0.125 µg/mL in coating buffer [45mM NaHCO_3_, 18mM Na_2_H CO_3_]) was plated and incubated overnight at 4 °C. Following incubation, wells were washed with 0.5% PBST, and blocking buffer of 5% BSA was added 200 µL per well and incubated for 1 hour at 37 °C. Next, wells were washed again, and 100 µL of patient serum diluted at 1:200 was added in duplicate. A standard from a deidentified patient with known CD [14] was serially diluted with an initial dilution of 1:200 and subsequent dilutions of 1:2 for a total of 7 dilutions and plated in triplicate with 3 additional buffer-only wells. Following incubation at 37 °C for 1 hour, wells were again washed, and 100 µL of goat anti–human IgG alkaline phosphatase (109-055-003; Jackson Laboratories) diluted at 1:2500 was added and incubated for 1 hour at 37 °C. Wells were again washed, and 100 µL of developer solution was added in a concentration of 1 phosphatase tablet (S0942; Sigma) per 5 mL of sodium carbonate buffer (CUS-0242; KD Medical). Upon addition of the developer solution, absorbance values were read on an Epoch2 microplate reader (BioTek) after 30 minutes at 405- to 410-nm wavelengths. Plotted curves were automatically standardized to the plated standard curves with Gen5 software (BioTek).

### Serum Samples and Data Analysis

Human serum samples were obtained from a previous study under an approved institutional review board protocol (H-37138; Baylor College of Medicine) and deidentified of all identifying information. Samples from Argentina—10 Chagas-positive (seropositive and PCR positive) and 32 Chagas-negative (seronegative and PCR negative)—were used to construct a receiver operator characteristic curve to identify the threshold of positivity (100% sensitivity, 100% specificity [14]) for both assays. These thresholds of positivity were then used to analyze clinical serum samples of unknown Chagas status from Ecuador, Guatemala, Honduras, Mexico, Colombia, and Brazil. Signal-to-background ratio was calculated as the ratio of the concentration of anti–*T cruzi* IgG in Chagas-positive and Chagas-negative samples. Statistical analysis, including Fisher exact, Kruskal-Wallis, Cohen κ, and Wilcoxon paired testing, was conducted in Prism version 10.3.0 (GraphPad).

## RESULTS

### Principle of the Magneto-Immunoassay

**Figure 1.**
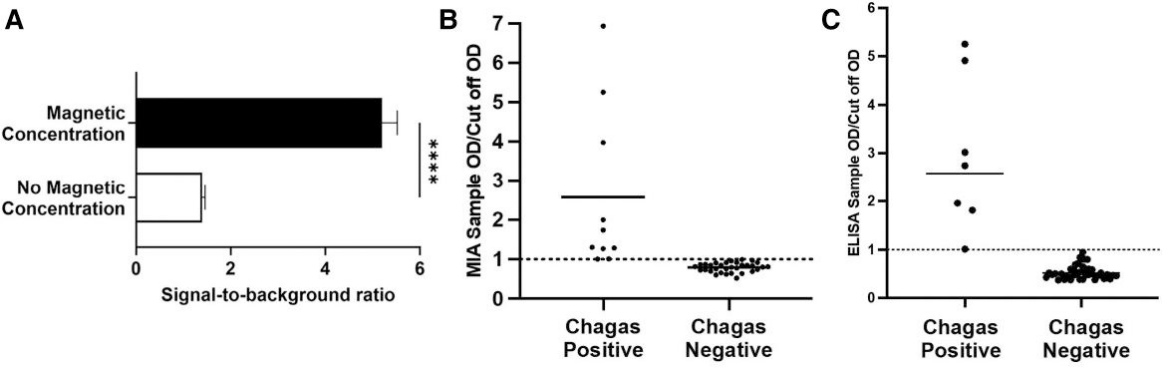
Analytical performance of the magneto-immunoassay. *A*, Signal-to-background ratios generated from 1000×-diluted Chagas-positive human sera with 1 minute of magnetic concentration (dark bar) or a 50-minute incubation without magnetic concentration (light bar). Each bar represents the mean ± SD of 3 replicates (*P* < .0001). Cutoff values for discriminating between Chagas-positive and Chagas-negative cases based on receiver operating characteristic curves generated with serum samples from Argentina for (*B*) the magneto-immunoassay and (*C*) the Tc24-based ELISA. Each point represents the absorbance (OD, 450 nm) generated by a unique serum sample divided by the absorbance (OD, 450 nm) of the assay cutoff value. Abbreviations: ELISA, enzyme-linked immunosorbent assay; MIA, magneto-immunoassay; OD, optical density.

The magneto-immunoassay employs magnetic nanoparticles to enhance the capture of anti–*T cruzi* IgG in the sample and accelerate the transport of DMB–*T cruzi* IgG conjugates, enabling the detection of low anti–*T cruzi* IgG concentrations while shortening the incubation times. During the initial incubation step, the serum sample is incubated with DMBs, allowing anti–*T cruzi* IgG in the sample to bind to the DMBs and form DMB–*T cruzi* IgG conjugates. The DMB–*T cruzi* IgG conjugates are separated by a magnetic rack and plated into a 96-well plate. The microwell plate is placed on a magnetic stage, causing the DMB–*T cruzi* IgG conjugates to rapidly concentrate on the bottom of the microwells and bind the capture antibody. TMB substrate is added to the microwells, which reacts with the HRP on the DMBs, resulting in a color change that is proportional to the anti–*T cruzi* IgG concentration. The use of DMBs and magnetic concentration enables the target analyte to be detected at concentrations 3-fold lower than without the use of DMBs and magnetic concentration (Figure 1*A*). This is due to the high surface area of DMBs, which provides multiple binding sites for the target analyte, and the presence of additional HRP molecules. Furthermore, DMBs are rapidly concentrated on the bottom of the microwells within minutes, which significantly increases binding kinetics and minimizes nonspecific binding due to shorter incubation times, reducing the total assay time to <1 hour.

### Validation of the Magneto-Immunoassay by Testing Clinical Serum Samples

**Figure 2.**
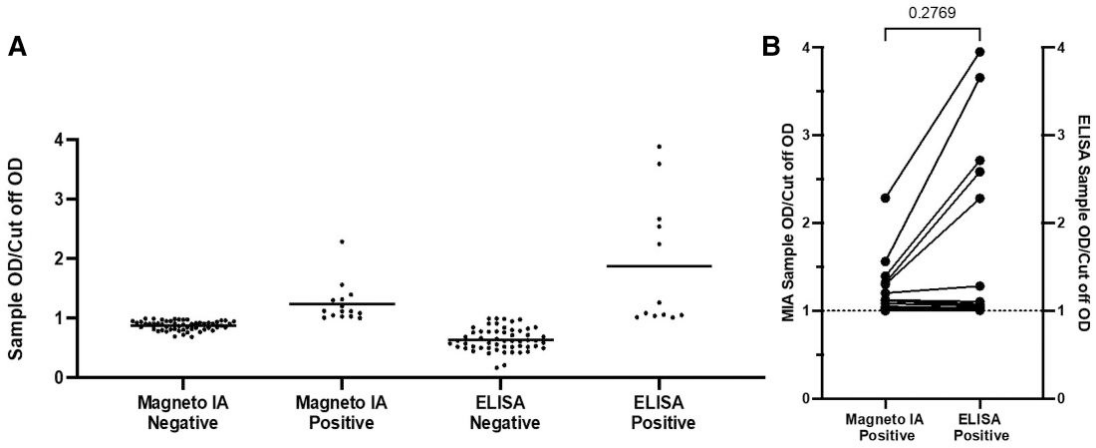
Validation of the magneto-immunoassay. *A*, Measurements of anti–*Trypanosoma cruzi* IgG in clinical serum samples of unknown Chagas status using the magneto-immunoassay and standard Tc24-based ELISA. Each point represents the absorbance (OD, 450 nm) generated by a unique serum sample divided by the absorbance (OD, 450 nm) of the assay cutoff value. Statistical significance was assessed by the Kruskal-Wallis test with significance defined as *P* < .0001. *B*, Sample OD/cutoff OD for 14 Chagas-positive samples generated by the magneto-immunoassay and the Tc24-based ELISA. Statistical significance was assessed by the Wilcoxon paired test with significance defined as *P* = .2769. Abbreviations: ELISA, enzyme-linked immunosorbent assay; IA, immunoassay; OD, optical density.

Thresholds of positivity for Chagas-positive cases were determined for the magneto-immunoassay and the Tc24-based ELISA with the 100% sensitivity and 100% specificity concentration value from their receiver operator characteristic curves (Figure 1*B* and 1*C*). Measurements of 60 serum samples of unknown Chagas status (10 samples from each of the following countries: Ecuador, Guatemala, Honduras, Mexico, Colombia, and Brazil) were performed using the magneto-immunoassay and Tc24-based ELISA. Both assays identified all of the Chagas-positive cases from 5 countries (Ecuador, Guatemala, Honduras, Colombia, and Brazil) and all of the Chagas-negative cases from all 6 countries (Figure 2*A*). The 2 assays equally detected Chagas-positive and Chagas-negative cases from the 42 Argentina samples (κ = 1.0, perfect agreement). However, the magneto-immunoassay identified 3 additional Chagas-positive cases from Mexico as compared with the Tc24-based ELISA, which were slightly below the threshold of positivity of the ELISA (κ = 0.8, substantial agreement). For 5 of the Chagas-positive cases, the Tc24-based ELISA generated higher detection signals relative to the magneto-immunoassay (Figure 2*B*). However, for the remaining 9 Chagas-positive cases, the Tc24-based ELISA generated lower detection signals relative to the magneto-immunoassay. Comparison of the magneto-immunoassay vs the Tc24-based ELISA revealed a sensitivity of 100% (95% CI, 75.75%–100%), a specificity of 94.83% (95% CI, 85.86%–98.49%), a positive predictive value of 80% (95% CI, 54.81%–92.95%), and a negative predictive value of 100% (95% CI, 93.47%–100%).

## DISCUSSION

We report a unique magneto-immunoassay for rapid and high-sensitivity detection of anti–*T cruzi* IgG in serum samples. Measurements of clinical samples from Chagas-endemic countries in Latin America encompassing all 6 *T cruzi* DTUs revealed the ability of the magneto-immunoassay to distinguish Chagas-positive from Chagas-negative cases based on anti–*T cruzi* IgG levels with better accuracy than a standard Tc24-based ELISA. We attribute the improved performance of the magneto-immunoassay to its ability to detect lower anti–*T cruzi* IgG levels and generate a lower background signal for Chagas-negative samples, both of which are due to the use of the DMBs and magnetic concentration. These attributes make the magneto-immunoassay particularly useful for analyzing serum samples with low anti–*T cruzi* IgG levels, as seen in patients with a weak adaptive immune response [15]. Furthermore, as Tc24 is highly specific to *T cruzi* and homologous across all *T cruzi* DTU subtypes, it can allow for the accurate detection of chronic CD from multiple geographic strains of *T cruzi*, thereby reducing the incidence of false-negative test results. This can decrease the morbidity and mortality associated with complications arising from untreated chronic infection and lower the transmission of CD in endemic regions. In addition to having the potential for offering high sensitivity and specificity, as well as high positive and negative predictive values, for the diagnosis of chronic CD, this assay is compatible with standard immunoassay protocols and plate readers, making it a promising diagnostic platform for use in Chagas-endemic regions. This work is aimed at comparing the performance of our magneto-immunoassay prototype with that of a standard Tc24-based ELISA for the detection of anti–*T cruzi* IgG in human serum. As such, the limitations of this study include a small sample size and the lack of comparison between the Tc24-based immunoassays and commercial Chagas tests, which are planned for future studies.

## Notes

***Author contributions.*** R. M. and P. B. L. designed the study. B. Z. produced and purified the Tc24 protein. K. L. S. and R. M. processed the samples and performed the assays. K. L. S., R. M., and P. B. L. performed data analysis. K. L. S., R. M., and P. B. L. wrote the manuscript. All authors reviewed the manuscript for scientific content and approved the final version.

***Financial support.*** This work was supported by the National Institutes of Health (grant number R21AI171477 to P. B. L. and R. M.).
